# Diets Rich in Olive Oil, Palm Oil, or Lard Alter Mitochondrial Biogenesis and Mitochondrial Membrane Composition in Rat Liver

**DOI:** 10.1155/2022/9394356

**Published:** 2022-02-21

**Authors:** Youzan Ferdiand Djohan, Massara Camara-Cissé, Gilles Fouret, Béatrice Bonafos, Bernard Jover, Jean-Paul Cristol, Charles Coudray, Christine Feillet-Coudray, Eric Badia

**Affiliations:** ^1^Laboratoire de Biochimie CHU, Université Félix Houphouët-Boigny, Cocody, Abidjan, Côte d'Ivoire; ^2^DMEM, INRA, Université Montpellier, Montpellier, France; ^3^PhyMedExp, Université Montpellier, INSERM U1046, CNRS, UMR 9214, Montpellier, France; ^4^Laboratoire de Biochimie, CHU-Lapeyronie, Montpellier, France

## Abstract

Palm oil (crude or refined) and lard are rich in SFA, while olive oil is rich in polyunsaturated fatty acids. SFA are considered harmful to health, while polyunsaturated fatty acids are beneficial to health. The aim of this study was to determine the effect of diets rich in crude PO, refined PO, OO, or lard on the mitochondrial membrane, the activity of mitochondrial respiratory chain complexes, and mitochondrial biogenesis. This was an experimental study in male Wistar rats fed a diet containing 30% of each oil. Rats had free access to food and water. After being fed for 12 weeks, animals were sacrificed and liver mitochondria were collected. This collection was used to determine membrane potential and ROS production, membrane phospholipid and fatty acid composition, citrate synthase activity and respiratory chain complex, cardiolipin synthase protein expression, and expression of selected genes involved in mitochondrial biogenesis. We found that diets rich in olive oil, palm oil, or lard altered mitochondrial biogenesis by significantly decreasing Pgc1*α* gene expression and altered the fatty acid composition of rat liver mitochondrial membrane PL.

## 1. Introduction

The most important role of mitochondria is the production of adenosine triphosphate (ATP) in the cell, which is used as an energy source. In addition to their role in cellular energy metabolism, mitochondria are also involved in cell signalling, differentiation and death, and in the control of the cell cycle and cell growth [1]. These processes in turn influence mitochondrial biogenesis [2, 3]. Their dysfunction has been associated with several human diseases [4]. The inner and outer mitochondrial membranes are made up of phospholipids (PL) [5]. The fatty acid composition of PL is diet dependent and influences mitochondrial membrane functions [6]. Insulin resistance (IR) alters mitochondrial function through (i) decreased expression of the gene encoding peroxisome proliferator activator receptor *γ* coactivator-1*α* (PGC1*α*) with decreased mitochondrial density [7], (ii) decreased oxidative phosphorylation [8], and (iii) abnormalities in mitochondrial morphology and impaired mitochondrial respiration and ATP synthesis [8].

High-fat diets (HFD) that favor their accumulation in the form of diacylglycerol, triacylglycerol, and ceramides are directly associated with IR [9, 10]. In addition, studies have highlighted the role of saturated fatty acids (SFA), particularly palmitic acid, in the development of IR [11, 12]. It should be noted that the majority of studies on fatty acids (saturated or unsaturated) use cafeteria diets, i.e., composed of a mixture of fatty acids. The particularity of this study lies in the fact that it uses vegetable oils with all their components (SFA, MUFA, PUFA, vitamins, polyphenols, etc.). Palm oil (PO) is rich in SFA, which represent about 50% of total fatty acids [13, 14]. SFA content of PO is close to that of lard, which is composed of 45% SFA [15]. Olive oil (OO) is very rich in unsaturated fatty acids [16] and polyphenols [17]. These properties give it nutritional benefits [18, 19].

However, it is not known whether excess consumption of crude PO, refined PO, OO, or lard alters the physicochemical properties of the mitochondrial membrane, the activity of mitochondrial respiratory chain complexes, and mitochondrial biogenesis.

The aim of this study was to determine the effect of diets rich in crude PO, refined PO, OO, or lard on the mitochondrial membrane, the activity of mitochondrial respiratory chain complexes, and mitochondrial biogenesis.

## 2. Materials and Methods

### 2.1. Animals and Diets

Forty young male Wistar rats (Charles River, L'Arbresle, France) aged 6 weeks at the beginning of experiments were housed under conditions of constant temperature (20–22°C), humidity (45–50%), and a standard dark cycle (20.00–08.00 h) with free access to food and water. Rats were randomised into five groups of eight animals and fed for 12 weeks either a standard rat chow diet (control diet) or one of the four HFD. In control diet, 11% of the energy was given by fat (5% soybean oil), whereas in HFD, 56% of the energy was provided by fat intake [20]. The fat-enriched diets consisted of 2.5% (w/w) of soybean oil and 30% (w/w) of crude PO, refined PO (Sania Cie, Abidjan, Côte d'Ivoire), OO (virgin olive oil, bought in a supermarket), or lard (Alva, Rezé, France). The detailed composition of these experimental diets is given in Table 1. After being fed for 12 weeks, rats were anaesthetized with 1% pentobarbital (50 mg/kg) (Ceva Santé Animale, Libourne, France).

The experiments complied with the guidelines for the care and use of laboratory animals (National Academies Press (US), 8th edition, 2011), and all procedures were approved by the local ethical committee (reference CEEA-LR-12002, Montpellier, France).

### 2.2. Rat Sacrifice and Sampling

Rats were anaesthetized with 1% pentobarbital (50 mg/kg ip) (Ceva Santé Animale, Libourne, France). The rat liver was quickly removed, rinsed in 0.9% NaCl solution, cut into different parts that were immediately frozen in liquid nitrogen, and then kept at −80°C until analysis, except one part that was used for the immediate isolation of fresh mitochondria.

Mitochondria were isolated from the fresh liver by the differential centrifugation techniques previously described [21]. One aliquot of the mitochondrial suspension was used for mitochondrial membrane potential and reactive oxygen species (ROS) production. One aliquot was frozen at −80°C for other measurement. Protein content was determined according to Bradford with bovine serum albumin as the standard [22].

### 2.3. Liver Triglycerides and Histological Lipid Analyses

Liver samples were homogenized in NaCl (9 g/L) and Triton X-100 (0.1%). The liver contents of triglycerides were quantified on the liver homogenates by enzymatic methods using the triglycerides PAP kit (bioMérieux, France).

To visualise hepatic lipid accumulation, Oil Red O stainings were applied. Frozen liver 5 *μ*m sections were stained with Oil Red O. Liver sections were observed under an AxioImager Z1 Zeiss microscope. Images were acquired using the AxioImager software driving a CoolSNAP CCD camera on 5 fields (20X) per section. The staining area was measured in a given field and expressed as a percentage of the total area within the field. For each group, liver samples from 6 to 8 rats were prepared and stained.

### 2.4. Liver Mitochondrial Membrane Characteristics and Enzyme Activities

Liver mitochondrial membrane potential and ROS production were assessed as previously described [23, 24]. The enzymatic activity of citrate synthase (CS) in the liver and mitochondrial respiratory complexes (complex I, complex II + III, and complex IV) in liver mitochondria were determined spectrophotometrically as previously described [25]. In brief, the activity of CS was measured by following the color of 5-thio-2-nitrobenzoic acid, which is generated from 5,5′-dithiobis-2-nitrobenzoic acid present in the reaction of citrate synthesis, and caused by the deacetylation of acetyl-CoA. Complex I activity was measured spectrophotometrically at 600 nm during 45 s by following the reduction of 2,6-dichloroindophenol by electrons accepted from decylubiquinol, which itself reduced after oxidation of NADH by complex I [26]. Complex II activity was measured spectrophotometrically at 600 nm by following the reduction of 2,6-dichloroindophenol by the succinate during 120 s. Complex II + III activities were measured spectrophotometrically by following the oxidation of cytochrome c at 550 nm during 90 s [27]. Cytochrome c oxidase (complex IV) activity was measured spectrophotometrically by following the oxidation of reduced cytochrome c at 550 nm during 30 s [28].

### 2.5. Analysis of Phospholipids in Liver Mitochondria

Mitochondrial membrane phospholipids were analysed as previously described [21]. Liver mitochondrial suspensions were extracted using a mixture of chloroform-methanol (2 : 1; v/v) according to Folch et al. [29] in the presence of 50 mg/l of butylated hydroxytoluene to prevent lipid oxidation. Phosphorus was quantified on Folch extracts of mitochondrial suspensions in order to determine total phospholipid quantity as previously described [21,30]. In brief, mitochondrial lipid extracts were automatically applied on silica gel 60 HPTLC plates (250 *μ*m, 20 × 10 cm), pretreated with 2-3% (w/v) boric acid in ethanol (100%), on a 4 mm band width using the ATS4 apparatus (CAMAG). The lipid spots' development was performed with methanol-acetic acid-pentane-chloroform (15 : 10 : 30 : 45, by vol.), which allowed the separation of phospholipids and neutral lipids on a 60-mm total migration distance. The scanning of the plates was carried out using a TLC scanner 3 (CAMAG) operating in the reflectance mode. The plates were scanned at 715 nm after dipping in a solution of Blue Spray (Sigma) (Blue Spray-4.2 M H_2_SO_4_-acetone, 1 : 2:3, by vol.) and heating for 3 min at 55°C. The different classes of phospholipids (sphingomyelin = SM, lysophosphatidylcholine = LPC, phosphatidylcholine = PC, phosphatidylinositol = PI, phosphatidylserine = PS, phosphatidylethanolamine = PE, and cardiolipin = CL) were finally identified by comparing their retention factor with authentic standards and quantified using calibration curves of the same standards.

Liver mitochondrial membrane phospholipid fatty acid analysis was determined as previously described [21, 31]. Fatty acid composition of mitochondrial phospholipids was compared in terms of the percentage content of various fatty acids.

### 2.6. Liver Lipid Mitochondrial Desaturase Indices and Unsaturation Index

Liver lipid mitochondrial desaturase indices and unsaturation index were determined as previously described [21]. In brief, desaturase indices are based on the ratio of product to precursor of individual fatty acids for a given desaturation reaction, so these indices were calculated as follows: desaturase Δ9 (16: 1*n*-7/16 : 0); desaturase Δ6 (18: 3*n*-6/18: 2*n*-6); and desaturase Δ5 (20 : 4(*n*-6)/20 : 3(*n*-6)) [32]. The unsaturation index (UI) was calculated from the relative percentage of each type of MUFA and PUFA multiplied by the number of double bonds present in the molecule [25].

### 2.7. Western Blotting Was Performed as Previously Described [33]

In brief, whole-cell protein lysates were prepared in the lysis buffer: 20 mM Tris pH 8, 50 mM DL-Dithiothreitol, 150 mM NaCl, 2 mM EDTA, 1% Triton X-100, 0.1% SDS, 1 mM PMSF, 1 mM orthovanadate, and 1% (v/v) of antiprotease cocktail (Sigma). Proteins were resolved by SDS-PAGE and then transferred to nitrocellulose membranes using refrigerated Tris-glycine transfer buffer at 20 V overnight. Membranes were blocked in 5% nonfat milk in PBS (without Tween) for 1 h at room temperature. Then, membranes were incubated overnight with primary antibody of CL synthase (CLS) (Abcam, France) and tubulin (Sigma-Aldrich, France) in blocking buffer. After washing with PBS/Tween under gentle agitation, membranes were incubated for 45 min in the dark with the fluorescent-labeled secondary antibodies and finally quantified using the Odyssey infrared imaging system (LI-COR, Lincoln, USA).

### 2.8. Liver mRNA Expression

Real-time quantitative PCR (RT-qPCR) was used to measure the expression of several genes involved in liver mitochondrial biogenesis. Total RNA was extracted with TRIzol reagent (Invitrogen Life Technologies, Cergy Pontoise, France). Reverse transcription reactions were performed on 500 ng total RNA using a reverse transcription Takara Kit (Takara Bio Europe, France). The mRNA expressions of target genes (mitochondrial transcription factor A (Tfam), peroxisome proliferator activator receptor *γ* coactivator-1*α* (Pgc1*α*), and nuclear respiratory factor-1 (Nrf1)) were determined by RT-qPCR using the LightCycler^®^ 480 SYBR Green I Master (Roche Applied Science, France). The results were normalized to Rplp0 gene. The primer sequences used for real-time RT-PCR are the following: Rplp0 forward CACTGGCTGAAAAGGTCAAGG, Rplp0 reverse GACTTGGTGTGAGGGGCTTA; Pgc1*α* forward TGTGGAACTCTCTGGAACTGC, Pgc1*α* reverse GCCTTGAAAGGGTTATCTTGG; Tfam forward AGCTAAACACCCAGATGCAAA, Tfam reverse TCAGCTTTAAAATCCGCTTCA; Nrf1 forward TTATTCTGCTGTGGCTGATGG, Nrf1 reverse CCTCTGATGCTTGCGTCGTCT.

### 2.9. Statistical Analysis

The results were expressed as mean values ± SD, *n* = 7-8 animals per group. Statistical analysis was based on one-way ANOVA followed by a Tukey-Kramer multiple comparisons test. When statistical variances were unequal, a Welch test was performed. The limit of statistical significance was set at *p* < 0.05. The group mean values with different letters (a, b, c, and d) are significantly different. Statistical analyses were performed using the StatView program (SAS Institute, Cary, NC, USA).

## 3. Results and Discussion

### 3.1. Hepatic Lipid Infiltration

**Figure 1:**
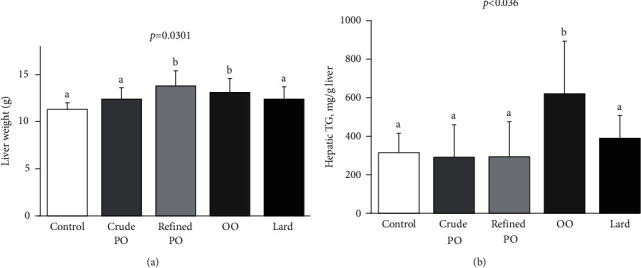
Effect of high dietary intake of palm oil, olive oil, and lard on (a) liver weight and (b) liver triglycerides. The results are expressed as mean values ± SD, *n* = 7-8 animals per group. The limit of statistical significance was set at *p* < 0.05. Bar graphs with different letters (a, b, and c) are significantly different. OO, olive oil; PO, palm oil; TG, triglycerides.

All HFD promoted an increase in liver weight (crude PO + 9%, lard + 9%, refined PO + 22%, and OO + 16%). Compared to control diet, these increases were significant with refined PO and OO diets (*p*=0.0301) (Figure 1(a)). However, high dietary intake of OO did significantly increase liver TG levels (*p* < 0.036), whereas the high intake of both PO and lard did not (Figure 1(b)).

**Figure 2:**
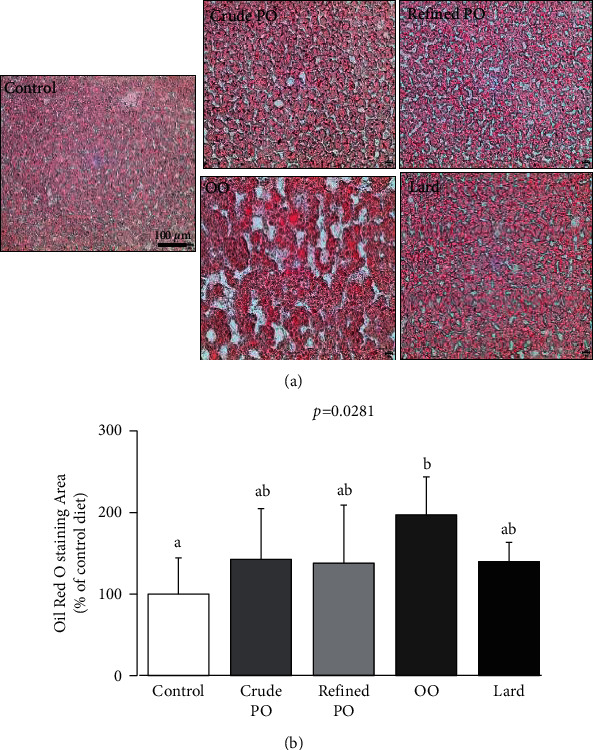
Effect of high dietary intake of palm oil, olive oil, and lard on hepatic lipid accumulation and steatosis. (a) Liver histology sections from a representative rat of each group, magnification 200x; (b) histograms represent liver lipid area quantification. The results are expressed as mean values ± SD, *n* = 7-8 animals per group. The limit of statistical significance was set at *p* < 0.05. Bar graphs with different letters (a, b, and c) are significantly different. OO, olive oil; PO, palm oil.

In addition to the high hepatic TG level, the liver histological lipid staining with Oil Red O, reflecting hepatic steatosis, showed significant differences (*p* < 0.0281) among the studied groups (Figure 2). More specifically, compared to control diet, the high intake of OO did increase the liver lipid area, whereas the high intake of PO and lard did not.

The accumulation of hepatic lipid induced by the consumption of OO has been demonstrated in many studies. Arbones-Mainar et al. [34] found the same results in mice fed for 10 weeks with three HFD containing 20% fat. This study compared the effects of three vegetable oils (PO, low polyphenol OO, and high polyphenol OO) on the liver. These authors observed a significantly greater hepatic steatosis with OO diets than with PO diet. Ferramosca et al. [35] found a significant increase in triglycerides and esterified cholesterol in the liver of mice fed a diet containing 7.5% OO for 8 weeks compared to mice fed a diet containing 7.5% corn oil. Some studies carried out in rats have shown that the consumption of OO leads to an increase in hepatic lipid deposition that is generally higher than with other diets, whether they are low in fatty acid [36, 37] or high in fatty acid [38, 39]. The high richness of OO in MUFA, 60–70% [40], could explain the significant increase in hepatic triglycerides and esterified cholesterol induced by the olive oil diet. These results show that a high intake of OO favors a fat accumulation in the liver.

### 3.2. Effects of Diets on Mitochondrial Membrane

The study of membrane potential, ROS production, phospholipid, and fatty acid composition of the membrane made it possible to assess the effects of diets on mitochondrial membrane. No significant difference was observed between the diets concerning membrane potential and ROS production (Table 2).

Total phospholipid content in rat liver mitochondria was not modified whatever the diet. Refined PO promoted a significant (*p*=0.016) 10% decrease in membrane CL content compared to the control diet. Regarding the content of other membrane PL, no significant difference was observed between the diets (Table 3).

Mitochondrial membrane PL content found in control rats was consistent with many studies [33, 41]. PC and PE are the two main classes of membrane phospholipids and account for about 80% of total liver phospholipids. CL, a mitochondria-specific phospholipid [42], accounts for about 9% of total phospholipids in mitochondria homogenate. PC and PE play a key role in membrane function. PC tends to fluidise the membrane, while PE tends to stiffen it [33]. Therefore, the PC/PE ratio is a key regulator of membrane integrity/fluidity [43]. A decrease in this ratio alters the properties of the membrane and could therefore also alter mitochondrial function.

Contrary to many studies [25, 41, 44] that showed that HFD led to a decrease in the PC/PE ratio with an increase in CL content, no HFD in our study favored the decrease in PC/PE ratio and the increase in CL. According to these authors, the increase in CL would be a means of compensating for the decrease in the PC/PE ratio in order to maintain the properties of the membrane. Indeed, CL plays an important role in the stability of the physical properties of the membrane and mitochondrial cristae [45].

Despite the significant 10% decrease in CL content induced by refined PO, no HFD promoted increased ROS production or disruption of membrane potential. On the other hand, HFD in our study promoted a nonsignificant increase in the CP/PE ratio of 7.74% for refined PO, 7.14% for crude PO, 5.95% for OO, and 4.17% for lard (Table 3). In addition, all HFD promoted a significant (*p* < 0.001) decrease in Δ9-desaturase activity compared to the control (Table 4). These results suggest an absence of deleterious effects of HFD based on palm oil (crude or refined), olive oil, and lard on the physicochemical properties of mitochondrial membrane [41, 46].

In addition to the polar parts of PL, the fatty acid composition of PL also influences membrane properties. Thus, fatty acid composition of PL plays an important role in membrane fluidity due to the chain length, degree, and type of unsaturation of these fatty acids. More specifically, the SFA/MUFA ratio is inversely proportional to membrane fluidity [33].

A significant decrease (*p* < 0.01) was observed in the SFA/MUFA ratio compared to control diet with crude PO (−23.07%), refined PO (−32.82%), and OO (−55.38%) diets (Table 4).

These results show the beneficial effect of these three oils on membrane fluidity. Crude PO and refined PO diets promoted a significant (*p* < 0.01) decrease in the PUFA/SFA ratio compared to the control diet, by 40% and 42%, respectively. This suggests that palm oil diets (crude or refined) may be more effective in protecting the mitochondrial membrane from ROS than other diets, as a decrease in the PUFA/SFA ratio makes mitochondria less susceptible to oxidative stress and lipid peroxidation [47].

Δ6-desaturase catalyzes the initial desaturation (limiting step) of linoleic and a-linolenic acids. Through a sequence of desaturation and chain lengthening reactions, these two essential fatty acids form arachidonic, eicosapentaenoic, and docosahexaenoic acids, which are the main PUFA substituents of membrane phospholipids, which regulate cell membrane fluidity [48]. The significant decrease (*p*=0.045) of Δ6-desaturase activity in the lard diet compared to the control diet (Table 4) could lead to a long-term disturbance of membrane fluidity in the mitochondria of lard-fed rats due to the absence of arachidonic, eicosapentaenoic, and docosahexaenoic acids in the phospholipids of the mitochondrial membranes [48, 49].

As shown in Table 4, despite their high SFA content, PO and lard diets did not significantly increase SFA content of the mitochondrial membrane PL. On the other hand, all HFD favored, compared to the control diet, an increase in MUFA and a decrease in PUFA of the mitochondrial membrane PL. These variations were significant with crude PO, refined PO, and OO but not with lard (Table 4). EPA and DHA contents of mitochondrial membrane PL were significantly lower in all HFD compared with the control diet (Table 4). This change in the FA composition of mitochondrial membrane PL has been observed by other authors [23, 25, 50]. These results show that excess crude PO, refined PO, OO, and lard alters the composition of the mitochondrial membrane.

### 3.3. Effects of Diet on Mitochondrial Enzyme Activity

The diets caused disturbances in the activity of certain mitochondrial enzymes (Table 5). OO and lard diets significantly (*p*=0.023) reduced hepatic CLS protein expression by 48% and 62%, respectively, compared to the control diet. Complex II activity was significantly (*p*=0.028) decreased by 15% in OO diet compared to the control diet (Table 5).

CLS is an essential enzyme in the biosynthesis of CL [51]. Decreasing CLS protein expression could lead to a decrease in CL synthesis. CL is closely associated with mitochondrial proteins and is thought to be involved in their function. Indeed, CL is required for optimal activity of complex I [52, 53], complex III [52, 54], complex IV [55], and complex V [56].

The significant 10% decrease in mitochondrial membrane CL content induced by refined PO did not affect the activity of respiratory chain complex. On the contrary, a significant decrease in complex II activity with OO diet associated with decrease of CLS protein expression was observed.

These results show that palm oil diets (crude or refined) do not have deleterious effects on respiratory chain enzymes as no significant differences were observed between control diet and PO diets with respect to their effects on citrate synthase, CLS, and respiratory chain complex. Lard had a deleterious effect on CLS protein expression, while OO had a deleterious effect on respiratory chain complex II activity and CLS protein expression.

### 3.4. Effects of Diets on Mitochondrial Biogenesis

**Figure 3:**
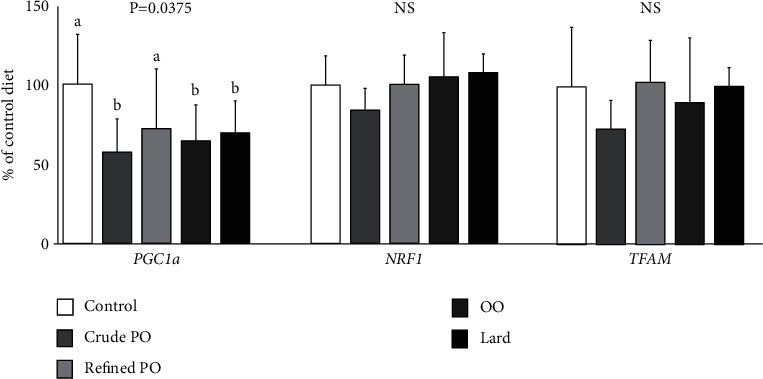
Expression of genes involved in liver mitochondrial biogenesis. Values are expressed as mean ± SD (*n* = 7-8). Histogram bars with different letters are significantly different. PO, palm oil; OO, olive oil; Pgc1*α*, peroxisome proliferator activator receptor *γ* coactivator-1*α*; Nrf1, nuclear respiratory factor 1; Tfam, mitochondrial transcription factor A.

No significant differences were observed between the diets concerning the gene expression of Nrf1 and Tfam. In contrast, all HFD repressed Pgc1*α* gene expression. This decrease in Pgc1*α* gene expression was significant (*p*=0.0375) with crude PO, OO, and lard diets compared to the control diet (Figure 3).

Many authors [57, 58] have shown that excessive fat intake promotes the decrease of Pgc1*α* gene expression.

Pgc1*α* is the main regulator of mitochondrial biogenesis, activating the expression of respiratory chain subunits and Nrf1, which is a potent stimulator of the expression of Tfam, polymerase RNA mitochondrial (Polrmt), and mitochondrial transcription factor B1 and B2 (Tfb1m and Tfb2m), which are factors involved in mitochondrial DNA replication and transcription [59, 60]. As such, a decrease in Pgc1*α* gene expression will result in a long-term disruption of mitochondrial function.

Although no significant differences in Nrf1and Tfam expression were observed between diets, the decrease in Pgc1*α* gene expression by HFD indicates that these diets impaired mitochondrial biogenesis.

Clearly, further work examining the metabolic role of excess vegetable oil in various pathological and nonpathological conditions/models, including more parameters, is warranted to learn more. Nevertheless, this study showed that PO and OO improved the fluidity of liver mitochondrial membrane. On the other hand, OO favored a fat accumulation in the liver and disturbed the activity of some enzymes of the respiratory chain. It appears that the increased intake of dietary fat, whatever its composition in saturated and unsaturated fatty acids, favored a disturbance of mitochondrial biogenesis. In light of these results, the normocaloric diet could help in the prevention of certain diseases related to mitochondrial dysfunction.

## Figures and Tables

**Table 1:** Diet composition (g/kg) on the basis of the AIN-93M diet formulation.

Ingredients	Control	Crude PO	Refined PO	OO	Lard
Casein	165	200	200	200	200
Cornstarch	443	234	234	234	234
Maltodextrin	144	80	80	80	80
Sucrose	100	53	53	53	53
Soybean oil	50	25	25	25	25
Crude palm oil	0	300	0	0	0
Refined palm oil	0	0	300	0	0
Olive oil	0	0	0	300	0
Lard	0	0	0	0	300
Cellulose	50	50	50	50	50
Mineral mix (AIN-93M)	35	42	42	42	42
Vitamin mix (AIN-93M)	10	12	12	12	12
L-cystine	2	2.4	2.4	2.4	2.4
Choline chloride	1.5	1.8	1.8	1.8	1.8
Total	1000	1000	1000	1000	1000

**Table 2:** Effect of diets on liver mitochondrial membrane potential and ROS production.

	Control	Crude PO	Refined PO	OO	Lard	*p*
Mitochondrial membrane potential						
Mitochondria (AU)	0.51 ± 0.3	0.49 ± 0.3	0.53 ± 0.1	0.53 ± 0.3	0.53 ± 0.3	NS
Mitochondria + substrate (AU)	0.68 ± 0.1	0.66 ± 0.2	0.70 ± 0.1	0.69 ± 0.3	0.68 ± 0.2	NS
Mitochondria + substrate + ADP (AU)	0.52 ± 0.1	0.52 ± 0.1	0.53 ± 0.1	0.52 ± 0.1	0.51 ± 0.1	NS

Mitochondrial ROS production						
Mitochondria (AU)	95 ± 20	101 ± 34	100 ± 28	87 ± 27	103 ± 34	NS
Mitochondria + substrate (AU)	150 ± 23	161 ± 33	159 ± 32	147 ± 29	152 ± 32	NS
Mitochondria + substrate + ADP (AU)	141 ± 24	153 ± 41	154 ± 37	138 ± 2	141 ± 33	NS

Values are expressed as mean ± SD (*n* = 7-8). ADP, adenosine diphosphate; AU, arbitrary units; OO, olive oil; PO, palm oil, ROS, reactive oxygen species.

**Table 3:** Effect of diets on liver mitochondrial membrane phospholipid (PL) composition.

	Control	Crude PO	Refined PO	OO	Lard	*p*
Total PL (nmol/mg protein)	228 ± 9.1	206 ± 12	236 ± 13	226 ± 7.4	230 ± 11	NS
LPC (%)	1.57 ± 0.29	1.74 ± 0.19	1.25 ± 0.18	1.43 ± 0.25	1.34 ± 0.27	NS
SM (%)	2.04 ± 0.06	1.89 ± 0.10	1.99 ± 0.13	2.16 ± 0.15	2.02 ± 0.19	NS
PC (%)	50.6 ± 0.56	51.9 ± 0.62	52.6 ± 0.59	51.8 ± 0.65	51.0 ± 0.63	NS
PI (%)	4.96 ± 0.17	5.54 ± 0.22	4.94 ± 0.36	5.00 ± 0.27	5.52 ± 0.32	NS
PS (%)	1.59 ± 0.21	1.20 ± 0.21	1.12 ± 0.26	1.15 ± 0.27	1.26 ± 0.28	NS
PE (%)	30.19 ± 0.32	28.88 ± 0.55	29.06 ± 0.47	29.30 ± 0.52	28.28 ± 0.55	NS
CL (%)	9.02 ± 0.26a	8.89 ± 0.29a	8.04 ± 0.32b	9.14 ± 0.26a	9.56 ± 0.32a	0.016
PC/PE	1.68 ± 0.03	1.80 ± 0.06	1.81 ± 0.05	1.78 ± 0.05	1.75 ± 0.05	NS

Values are expressed as mean ± SD (*n* = 7-8). On the same line, values with different letters are significantly different. (%), percentages in phosphorus of total phospholipids; CL, cardiolipin; LPC, lysophosphatidylcholine; OO, olive oil; PC, phosphatidylcholine; PE, phosphatidylethanolamine; PI, phosphatidylinositol; PL, phospholipid; PO, palm oil; PS, phosphatidylserine; SM, sphingomyelin.

**Table 4:** Effect of diets on liver mitochondrial phospholipid (PL) fatty acid (FA).

	Control	Crude PO	Refined PO	OO	Lard	*p*
Total SFA	39.1 ± 0.43a	41.1 ± 0.58a	39.9 ± 0.64a	30.9 ± 1.33b	38.8 ± 0.74a	<0.01
C16 : 0	25.4 ± 0.76a	27.4 ± 0.65b	27.3 ± 0.28b	20.4 ± 0.81c	22.6 ± 0.47c	<0.01
C18 : 0	12.5 ± 0.75ac	12.8 ± 0.87ac	11.6 ± 0.78ab	9.70 ± 1.34b	15.3 ± 1.00c	<0.01
Total MUFA	20.5 ± 1.22a	28.4 ± 1.73bd	30.8 ± 1.17b	37.9 ± 2.72c	24.7 ± 1.79ad	<0.01
C16: 1*n-*7	3.40 ± 0.35a	0.98 ± 0.17b	1.11 ± 0.18b	1.11 ± 0.13b	0.89 ± 0.07b	<0.01
C18: 1*n-*9	13.4 ± 0.92a	25.0 ± 1.69bd	27.1 ± 1.19b	33.3 ± 2.64c	20.8 ± 1.78d	<0.01
C18: 1*n-*7	3.10 ± 0.10a	1.62 ± 0.04b	1.60 ± 0.09b	2.64 ± 0.05c	2.50 ± 0.10c	<0.01
Total PUFA	40.4 ± 1.29a	30.5 ± 1.54b	29.3 ± 0.66b	31.2 ± 1.73b	36.5 ± 1.14a	<0.01
C18: 2*n-*6	18.4 ± 0.59a	11.1 ± 0.80b	10.8 ± 0.63b	13.5 ± 0.80c	14.4 ± 0.53c	<0.01
C18: 3*n-*6	0.42 ± 0.048a	0.21 ± 0.012b	0.21 ± 0.011b	0.24 ± 0.027b	0.21 ± 0.021b	<0.01
C18: 3*n-*3	0.78 ± 0.058a	0.14 ± 0.020b	0.14 ± 0.017b	0.24 ± 0.031b	0.38 ± 0.035c	<0.01
C20: 3*n-*6	0.29 ± 0.030	0.26 ± 0.051	0.32 ± 0.033	0.32 ± 0.042	0.33 ± 0.049	NS
C20: 4*n-*6	15.1 ± 1.07	14.3 ± 1.27	13.3 ± 0.68	12.7 ± 1.67	15.7 ± 1.20	NS
C20: 5*n-*3 = EPA	0.23 ± 0.034a	0.04 ± 0.006bc	0.03 ± 0.006b	0.05 ± 0.002bc	0.09 ± 0.006c	<0.01
C22: 5*n-*3	0.36 ± 0.023a	0.22 ± 0.021b	0.23 ± 0.014b	0.22 ± 0.014b	0.41 ± 0.047a	<0.01
C22: 6*n-*3 = DHA	4.19 ± 0.31a	2.74 ± 0.18b	2.64 ± 0.22b	3.02 ± 0.42b	3.15 ± 0.28b	<0.01
Total *n-*6	34.6 ± 1.03a	27.3 ± 1.41b	26.2 ± 0.57b	27.6 ± 1.35b	31.4 ± 0.90c	<0.01
Total *n-*3	5.72 ± 0.29a	3.19 ± 0.18b	3.09 ± 0.21b	3.57 ± 0.41b	5.13 ± 0.29a	<0.01
*n-*6/*n-*3	6.12 ± 0.20a	8.61 ± 0.36b	8.66 ± 0.53b	8.20 ± 0.68b	6.18 ± 0.22a	<0.01
SFA/MUFA	1.95 ± 0.12a	1.50 ± 0.12b	1.31 ± 0.08b	0.87 ± 0.11c	1.64 ± 0.16ab	<0.01
PUFA/SFA	1.04 ± 0.04a	0.74 ± 0.04b	0.73 ± 0.01b	1.01 ± 0.04a	0.94 ± 0.02a	<0.01
*n-*3/PUFA	0.141 ± 0.004a	0.105 ± 0.004b	0.106 ± 0.006b	0.113 ± 0.007b	0.140 ± 0.005a	<0.01
PL UI	152 ± 4.56a	133 ± 4.68bc	131 ± 2.19bc	141 ± 5.92ac	149 ± 3.88a	<0.01
Δ9-desaturase	0.132 ± 0.010a	0.035 ± 0.005b	0.041 ± 0.007b	0.054 ± 0.005b	0.040 ± 0.003b	<0.001
Δ6-desaturase	0.023 ± 0.002a	0.019 ± 0.002ab	0.020 ± 0.002a	0.018 ± 0.002ab	0.014 ± 0.001b	0.045
Δ5-desaturase	51.9 ± 2.35	52.8 ± 4.61	43.8 ± 4.29	41.4 ± 4.93	50.8 ± 4.53	NS

Values are expressed as mean ± SD (*n* = 7-8). On the same line, values with different letters are significantly different. Major fatty acids are represented, and the results are expressed as percentage of fatty acid of total phospholipids. DHA, docosahexaenoic acid; EPA, eicosapentaenoic acid; MUFA, monounsaturated fatty acids; OO, olive oil; PL, phospholipids; PO, palm oil; PUFA, polyunsaturated fatty acids; SFA, saturated fatty acids; UI, unsaturation index. Δ9-desaturase = 16 : 1*n-*7/16 : 0; Δ6-desaturase = 18 : 3*n-*6/18 : 2*n-*6; Δ5-desaturase = 20: 4*n-*6/20:3*n-*6. SFA/MUFA: SFA = % C16 : 0 + % C18 : 0 and MUFA = % C16 : 1 + % C18 : 1. PUFA/SFA: SFA = % C16 : 0 + % C18 : 0 and PUFA = % all PUFA. *n-*3/PUFA: total (*n-*3)/all PUFA.

**Table 5:** Variation in mitochondrial enzyme activity.

	Control	Crude PO	Refined PO	OO	Lard	*p*
Complex I (mU/mg protein)	1.56 ± 0.25	1.31 ± 0.16	1.40 ± 0.14	1.41 ± 0.14	1.42 ± 0.11	NS
Complex II (mU/mg protein)	1.85 ± 0.05^a^	1.74 ± 0.04^ab^	1.80 ± 0.08^a^	1.57 ± 0.07^b^	1.70 ± 0.07^ab^	0.028
Complex II + III (mU/mg protein)	0.078 ± 0.006	0.077 ± 0.005	0.094 ± 0.010	0.065 ± 0.007	0.069 ± 0.007	NS
Complex IV (mU/mg protein)	0.796 ± 0.086	0.767 ± 0.090	0.846 ± 0.129	0.819 ± 0.097	0.902 ± 0.058	NS
Citrate synthase (mU/mg protein)	96.9 ± 6.12	103 ± 7.12	101 ± 6.68	106 ± 5.12	104 ± 6.72	NS
CLS (WB)	1.00 ± 0.22^a^	0.64 ± 0.84^a^	0.63 ± 0.90^a^	0.52 ± 0.12^b^	0.38 ± 0.84^b^	0.023

Values are expressed as mean ± SD (*n* = 7-8). On the same line, values with different letters are significantly different. CLS, cardiolipin synthase; WB, western blot.

## Data Availability

The data used to support the findings of this study are included within the article.

## Abbreviations

AU: Arbitrary unit
CL: Cardiolipin
CLS: Cardiolipin synthase
CS: Citrate synthase
HFD: High-fat diet
IR: Insulin resistance
OO: Olive oil
PL: Phospholipid
PO: Palm oil
ROS: Reactive oxygen species
UI: Unsaturation index
WB: Western blot
WW: Wet weight.
